# A Critical Role of the Thy28-MYH9 Axis in B Cell-Specific Expression of the *Pax5* Gene in Chicken B Cells

**DOI:** 10.1371/journal.pone.0116579

**Published:** 2015-01-21

**Authors:** Toshitsugu Fujita, Fusako Kitaura, Hodaka Fujii

**Affiliations:** Chromatin Biochemistry Research Group, Combined Program on Microbiology and Immunology, Research Institute for Microbial Diseases, Osaka University, Suita, Osaka, Japan; Centre National de la Recherche Scientifique, FRANCE

## Abstract

Accumulating evidence suggests that Pax5 plays essential roles in B cell lineage commitment. However, molecular mechanisms of B cell-specific expression of Pax5 are not fully understood. Here, we applied insertional chromatin immunoprecipitation (iChIP) combined with stable isotope labeling using amino acids in cell culture (SILAC) (iChIP-SILAC) to direct identification of proteins interacting with the promoter region of the endogenous single-copy chicken *Pax5* gene. By comparing B cells with macrophage-like cells trans-differentiated by ectopic expression of C/EBPβ, iChIP-SILAC detected B cell-specific interaction of a nuclear protein, Thy28/Thyn1, with the *Pax5* 1A promoter. Trans-differentiation of B cells into macrophage-like cells caused down-regulation of Thy28 expression. Loss-of-function of Thy28 induced decrease in Pax5 expression and recruitment of myosin-9 (MYH9), one of Thy28-interacting proteins, to the *Pax5* 1A promoter. Loss-of-function of MYH9 also induced decrease in Pax5 expression. Thus, our analysis revealed that Thy28 is functionally required for B cell-specific expression of Pax5 via recruitment of MYH9 to the *Pax5* locus in chicken B cells.

## Introduction

Elucidation of molecular mechanisms of genome functions such as transcriptional regulation requires identification of components mediating the genome functions. To this end, we recently developed the locus-specific chromatin immunoprecipitation (ChIP) technologies to identify molecules interacting with a given genomic region of interest *in vivo* [1–8]. Locus-specific ChIP consists of insertional ChIP (iChIP) [1–4,8] and engineered DNA-binding molecule-mediated ChIP (enChIP) [5–7] using transcription activator-like (TAL) proteins and the clustered regularly interspaced short palindromic repeats (CRISPR) system [9]. Basically, locus-specific ChIP consists of locus tagging and affinity purification and can be combined with down-stream analyses such as mass spectrometry (MS) (iChIP-MS and enChIP-MS) to identify proteins, for example [2,5,7]. Identification of genome-interacting proteins by iChIP-MS and enChIP-MS is useful for elucidation of mechanisms of genome functions including transcription.

The *Pax5* gene encodes a transcription factor essential for B cell differentiation [10]. Disruption of the *Pax5* gene inhibits B cell differentiation from pro-B to pre-B cells in mice [11,12]. The *Pax5*-deficient pro-B cells can trans-differentiate into other lymphoid cell types [13,14], suggesting importance of Pax5 for B cell lineage commitment. Transcription mechanisms of the *Pax5* gene have been examined for more than a decade. It is reported that the intron 5 of mouse *Pax5* gene possesses enhancer regions, on which PU.1, IRF4, IRF8 and NF-κB function for the B cell-specific gene transcription [15]. The transcription factor EBF1 binds to the region 1.1 kbp upstream of the transcription start site (TSS) of the exon 1A, the B cell-specific first exon, and is required for Pax5 expression [15,16]. However, mechanisms of transcriptional regulation of the *Pax5* gene have not been fully understood.

In this study, we applied iChIP with stable isotope labeling using amino acids in cell culture (SILAC), a method of MS-based quantitative proteomics [17] (iChIP-SILAC) to direct identification of proteins interacting with the endogenous single-copy *Pax5* 1A promoter region in a chicken B cell line, DT40. By comparing a DT40-derived cell line with a macrophage-like cell line trans-differentiated by ectopic expression of C/EBPβ, the iChIP-SILAC analysis detected B cell-specific interaction of a nuclear protein, Thy28/Thyn1, with the *Pax5* 1A promoter. Loss-of-function of Thy28 induced decrease in Pax5 expression and recruitment of myosin-9 (MYH9), a Thy28-interacting protein, to the *Pax5* 1A promoter region. MYH9 was also required for Pax5 expression. Thus, our analysis revealed that Thy28 is functionally required for B cell-specific expression of Pax5 via recruitment of MYH9 to the *Pax5* locus in chicken B cells.

## Results

### iChIP-SILAC analysis to identify proteins interacting with the *Pax5* 1A promoter *in vivo*

**Figure 1:**
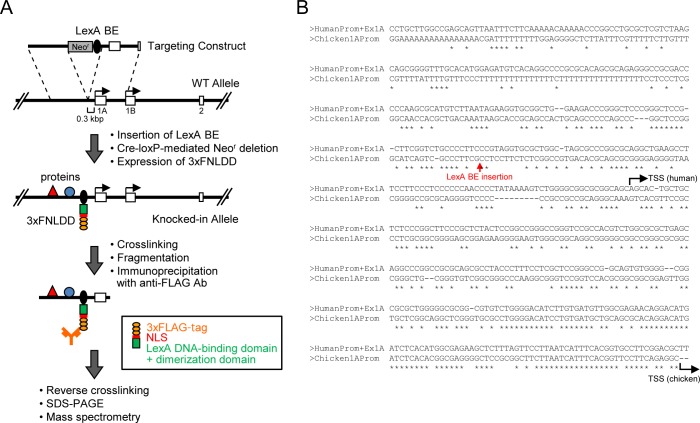
Scheme of iChIP analysis of the *Pax5* 1A promoter. **(A)** Scheme of iChIP analysis. 8 x LexA-binding elements (LexA BE) were inserted into 0.3 kbp upstream of the transcription start site (TSS) of the *Pax5* exon 1A. **(B)** The insertion site of LexA BE.

**Figure 2:**
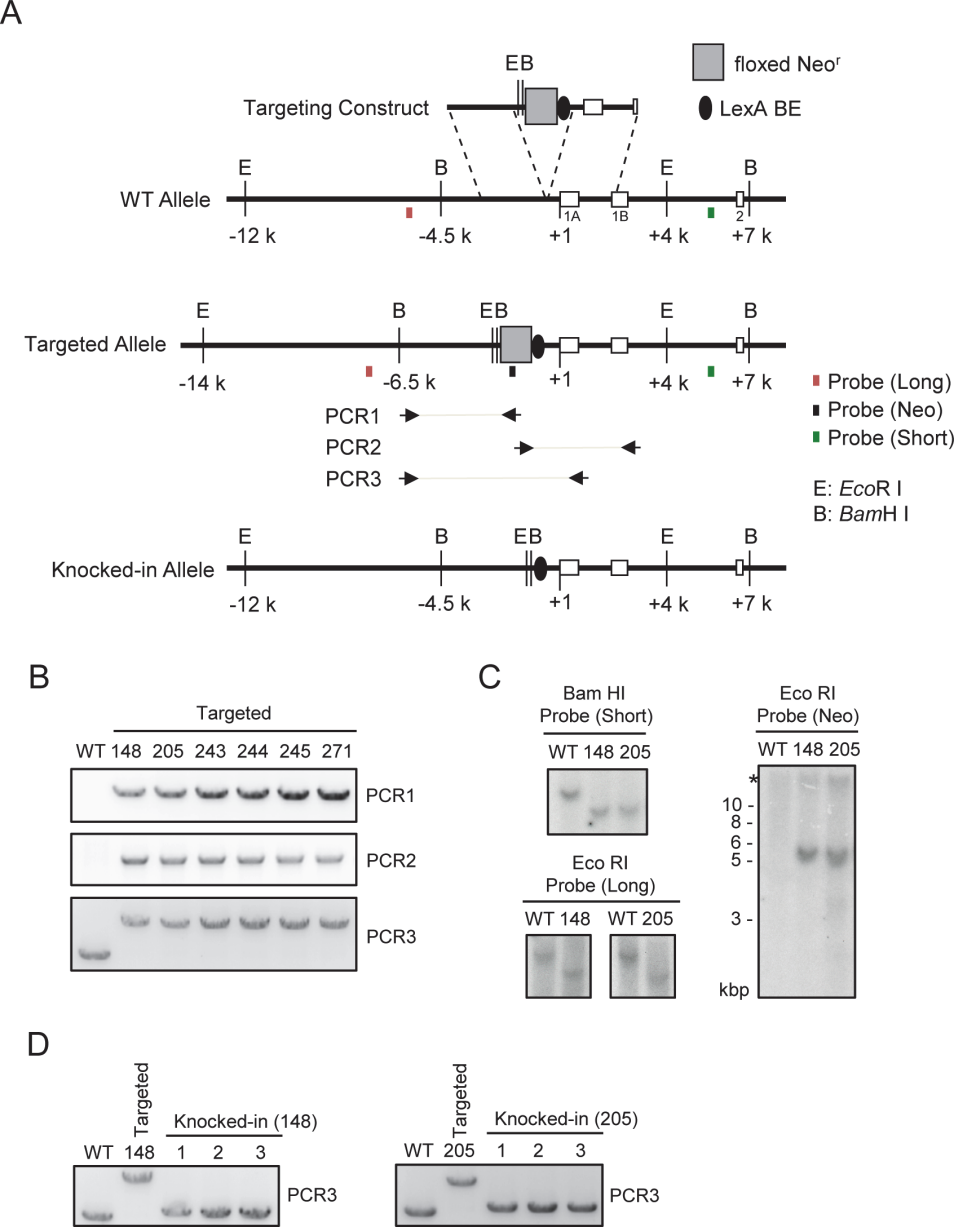
Knocking-in of LexA BE into the *Pax5* 1A promoter region. **(A)** Targeting strategy. **(B)** Genomic PCR to detect the targeted allele. **(C)** Southern blot analysis to detect the targeted allele. The asterisk indicates the position of non-specific bands. **(D)** Genomic PCR after Cre-mediated deletion of the floxed Neo^r^ gene to detect the knocked-in allele. The clone #205-2 was used as a representative knocked-in clone for expression of 3xFNLDD in Fig. 3A.

**Figure 3:**
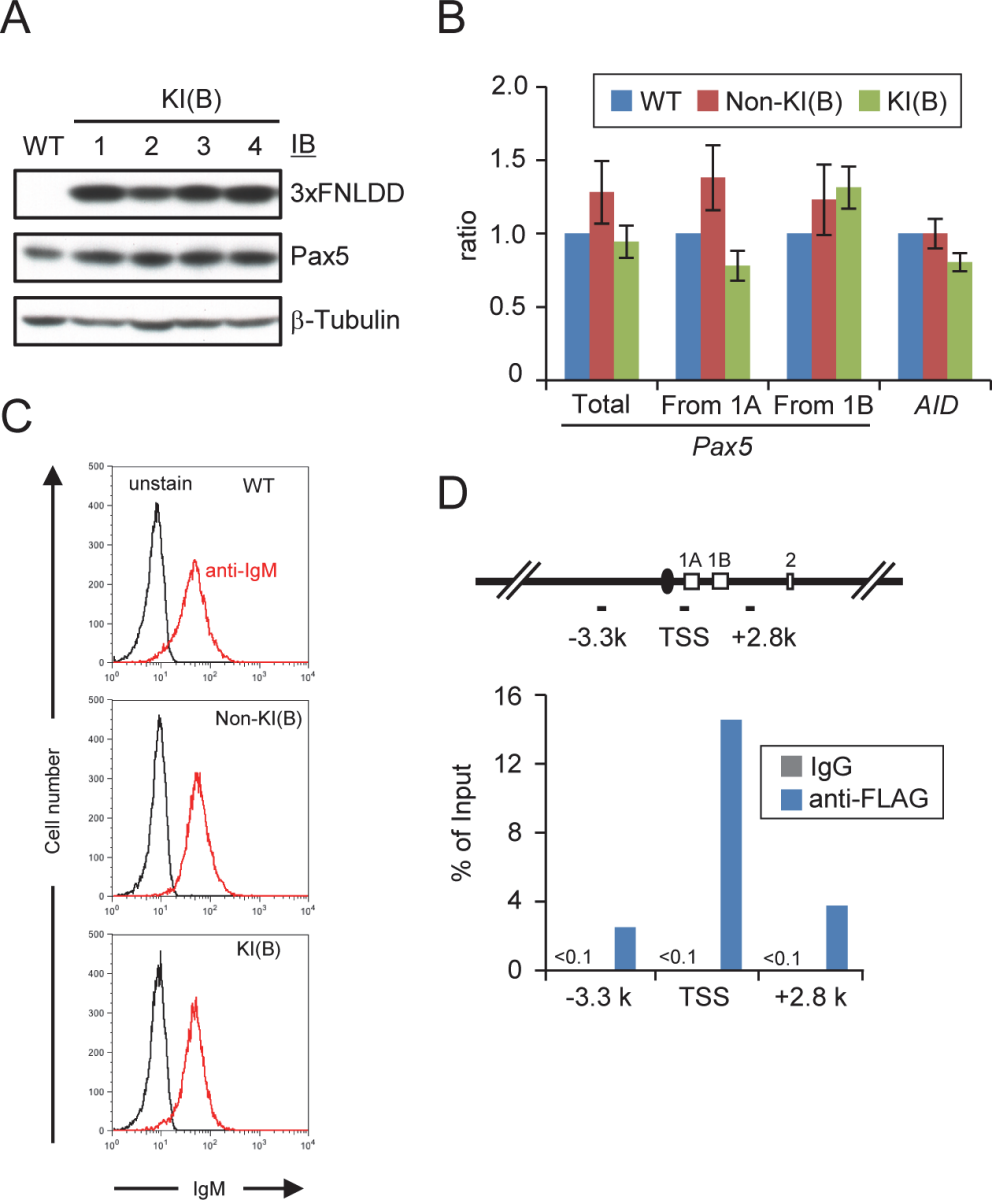
iChIP analysis of the *Pax5* 1A promoter. **(A)** Immunoblot analysis of 3xFNLDD and Pax5 proteins in KI(B) cell lines retaining LexA BE in the *Pax5* 1A promoter. **(B)** Expression of *Pax5* and *AID* mRNA in KI(B) #4 (hereafter referred simply as KI(B)). Expression levels of *Pax5* and *AID* mRNA were quantified by real-time RT-PCR and normalized to those of *GAPDH* mRNA (mean +/− SEM, n = 4). **(C)** Flowcytometric analysis of expression of cell surface IgM in KI(B). **(D)** Isolation of the *Pax5* 1A promoter region. The primer positions for real-time PCR and yield of iChIP analysis are shown in upper and lower panels, respectively. The data is a representative of two independent experiments.

To identify proteins interacting with a *Pax5* promoter region by iChIP (Fig. 1A), we inserted the 8 x LexA BE (0.16 kbp) into 0.3 kbp upstream of TSS of the exon 1A of *Pax5* gene [18] in a chicken B cell line, DT40 [19], by homologous recombination (Fig. 1A). DT40 shows high homologous recombination efficiency, which is advantageous for insertion of LexA BE for iChIP analysis. The *Pax5* locus is on the Z chromosome in chicken. Since DT40 is derived from female chicken retaining only one Z chromosome, the *Pax5* gene is a single-copy gene in DT40. Similar to human and mice, transcription of the chicken *Pax5* gene starts from both exons 1A and 1B in DT40 [18]. LexA BE was inserted into the *Pax5* 1A promoter region which is not conserved among species [18] (Fig. 1B), so that the insertion might not cause abrogation of *Pax5* transcription. Targeted integration was confirmed by genomic PCR as well as Southern blot analysis (Fig. 2A–C). Subsequently, the neomycin-resistance cassette was removed by transient expression of Cre recombinase (Fig. 2D). Next, the 3xFLAG-tagged LexA DNA-binding domain, 3xFNLDD [3], was expressed in the targeted clone, #205-2. Expression levels of Pax5 protein as well as *Pax5* mRNA from the exons 1A and 1B were comparable between the parental DT40, DT40 expressing 3xFNLDD (hereafter referred as Non-KI(B)) and knocked-in clones expressing 3xFNLDD (hereafter referred as KI(B) clones, and the clone #4 was used as a representative KI(B) clone for downstream experiments) (Fig. 3A, B), showing that the integration of LexA BE and expression of 3xFNLDD did not disrupt expression of the *Pax5* gene. Expression of markers of B cells such as *activation-induced deaminase (AID)* and IgM was also retained in these clones (Fig. 3B, C). Thus, the established clones maintained B cell phenotype. Next, we performed iChIP using anti-FLAG Ab to isolate the *Pax5* 1A promoter region. The yield of iChIP was 15% of input for a representative KI(B) clone (Fig. 3D), showing efficient isolation of the target region by iChIP.

**Figure 4:**
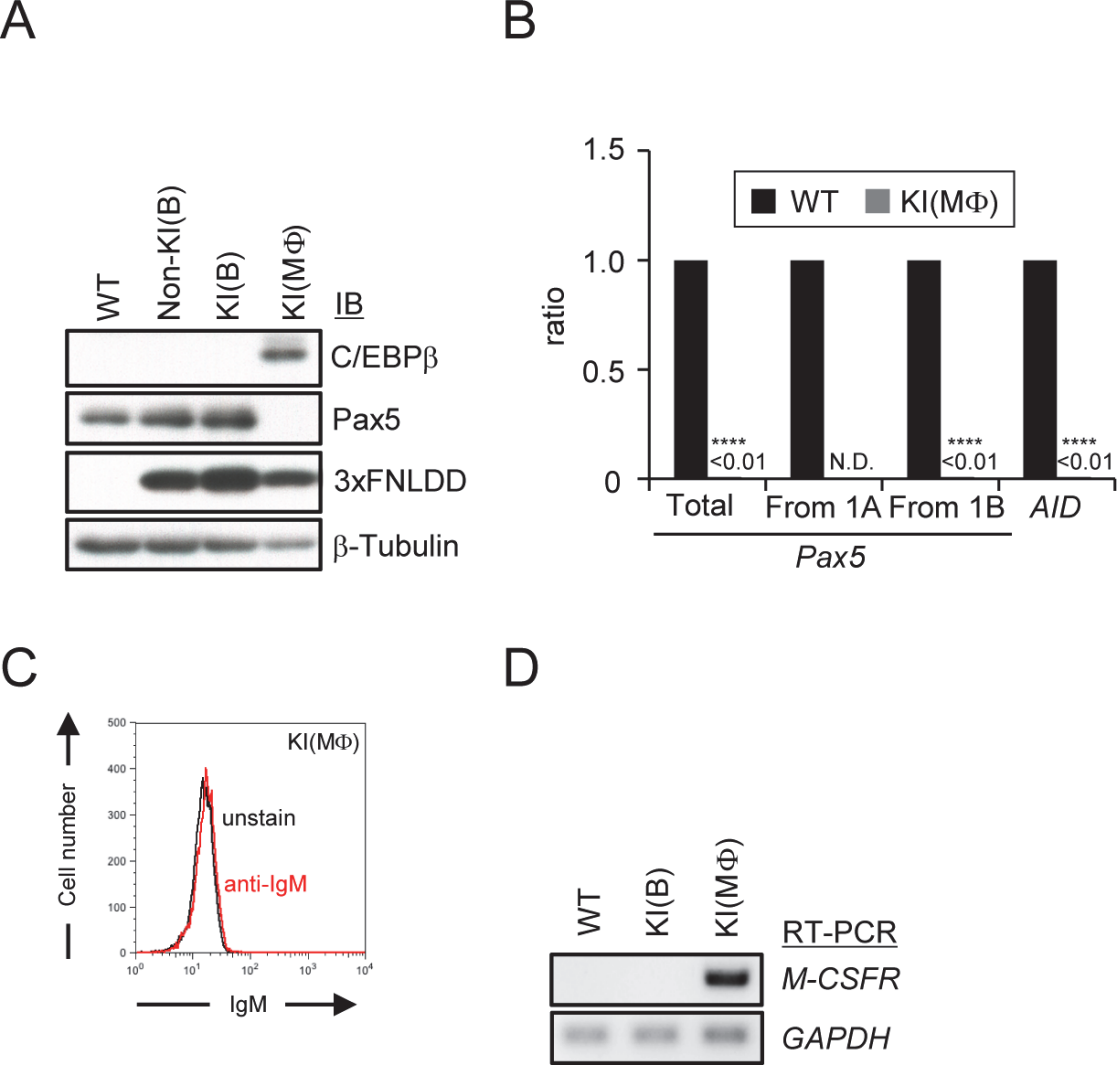
Trans-differentiation of KI(B) into macrophage-like cells by ectopic expression of C/EBPβ. **(A)** Immunoblot analysis of ectopically-expressed C/EBPβ, Pax5, and 3xFNLDD proteins. **(B)** Down-regulation of *Pax5* and *AID* mRNA by ectopic expression of C/EBPβ. Expression levels of *Pax5* and *AID* mRNA were quantified by real-time RT-PCR and normalized to those of *GAPDH* mRNA (mean +/− SEM, n = 3). p-values calculated between WT and KI(MΦ) are shown (****: p < 0.0001). N.D.: not detected. **(C)** Loss of expression of IgM by ectopic expression of C/EBPβ. **(D)** Expression of the *M-CSFR* gene by ectopic expression of C/EBPβ.

Identification of proteins interacting with the *Pax5* 1A promoter in a B cell-specific manner requires comparison of the interacting proteins in B cells as well as in different cell types. It has been shown that the ectopic expression of the transcription factor C/EBPα or β induces trans-differentiation of mouse mature B cells into macrophage-like cells [20]. To establish a non-B cell line for iChIP analysis, we attempted to induce trans-differentiation of KI(B) into macrophage-like cells by ectopic expression of C/EBPβ. An expression construct of chicken C/EBPβ cDNA was transfected into KI(B). In the representative clone stably expressing C/EBPβ (hereafter referred as KI(Φ)), expression of Pax5 protein as well as *Pax5* mRNA became undetectable (Fig. 4A, B). In addition, expression of *AID* and IgM was lost (Fig. 4B, C). On the other hand, markers of macrophage such as *macrophage-colony stimulating factor receptor (M-CSFR)* became detectable in KI(MΦ) (Fig. 4D). Thus, KI(MΦ) lost B cell phenotype and trans-differentiated into macrophage-like cells. KI(MΦ) was used as the negative control cell line for iChIP analysis.

**Figure 5:**
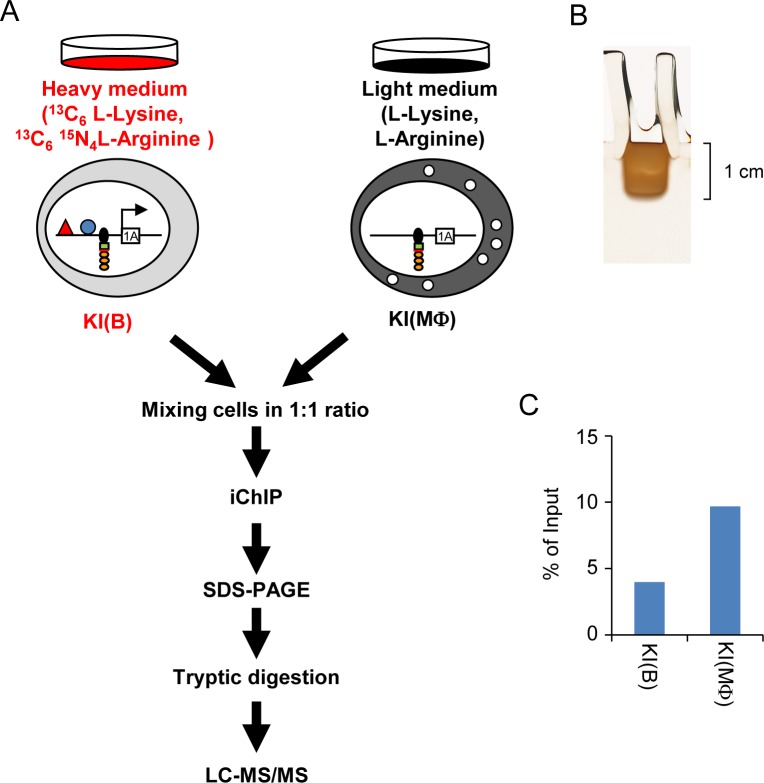
iChIP-SILAC. **(A)** Scheme of iChIP-SILAC. **(B)** SDS-PAGE and silver staining. The stained regions were equally divided into 5 parts (2 mm height each), excised, and subjected to in-gel tryptic digestion. The digested peptides were analyzed in LC-MS/MS. **(C)** iChIP efficiency with KI(B) and KI(MΦ) cells. After iChIP, iChIP efficiency was evaluated by amplification of the region adjacent to the LexA BE insertion site (“+0.2 k” in Fig. 3D) by real-time PCR and shown as ratio. The raw value of SILAC “Heavy/Light” in S1 Table was normalized to the iChIP efficiency and shown as normalized value in Table 1.

Next, we performed iChIP-SILAC to identify proteins interacting with the *Pax5* 1A promoter region in a B cell-specific manner. 5 × 10^7^ of each clone (KI(B) and KI(MΦ)) were subjected to iChIP-SILAC analysis (Fig. 5). We detected a list of proteins interacting with the *Pax5* 1A promoter (Tables 1 and S1 Table). The Heavy/Light value more than 1.00 shows that the identified proteins were detected more abundantly from the KI(B) sample than the negative control.

**Table 1:** Proteins identified by iChIP-SILAC (top 10).

**Proteins**	**Heavy/Light^#^**
Visual system homeobox 1	6.53
Thy28 (Thymocyte nuclear protein 1)	3.92
Histone H1.10	1.77
Tyrosine-protein kinase BTK	1.76
Acidic leucine-rich nuclear phosphoprotein 32 family member B	1.71
Asparagine synthetase [glutamine-hydrolyzing]	1.66
Histone H2B 7	1.64
Histone H1.11R	1.53
Macrophage migration inhibitory factor	1.52
Alpha-enolase	1.49

All of identified proteins, identified peptides, and raw Heavy/Light value are shown in S1 Table.

^#^Raw Heavy/Light value was normalized to iChIP efficiency of the *Pax5* 1A promoter region (Fig. 5C) and shown as normalized value in this table.

### Proteins interacting with the *Pax5* 1A promoter in a B cell-specific manner

In the list of proteins associated with the *Pax5* 1A promoter in a B cell-specific manner, we detected non-receptor type protein tyrosine kinase, Btk, which plays essential roles in signal transduction from B cell receptor (BCR) and development of B cells [21,22]. Although Btk is associated with the BCR complex on the plasma membrane, it has been shown that Btk is also localized in the nucleus and involved in transcriptional regulation [23,24]. The role of nuclear Btk in Pax5 expression would be an interesting future issue. We also detected histone variants and a histone chaperon (acidic leucine-rich nuclear phosphoprotein 32 family member B) [25]. It is possible that constituents of nucleosome in the *Pax5* 1A promoter might be different in B cells and non-B cells. In the list, VSX1 (visual system homeobox 1) and Thy28 showed highest SILAC Heavy/Light scores. Thy28 (also known as Thyn1) is a nuclear protein conserved among species, and expression levels of cThy28 are high in the bursa of Fabricius [26], which is the organ for B cell development in chicken. In contrast, expression levels of VSX1 are confined in the retina and spinal cord [27]. Therefore, we proceeded to analyze the function of Thy28 in the expression regulation of the *Pax5* gene.

### Thy28 regulates Pax5 expression

**Figure 6:**
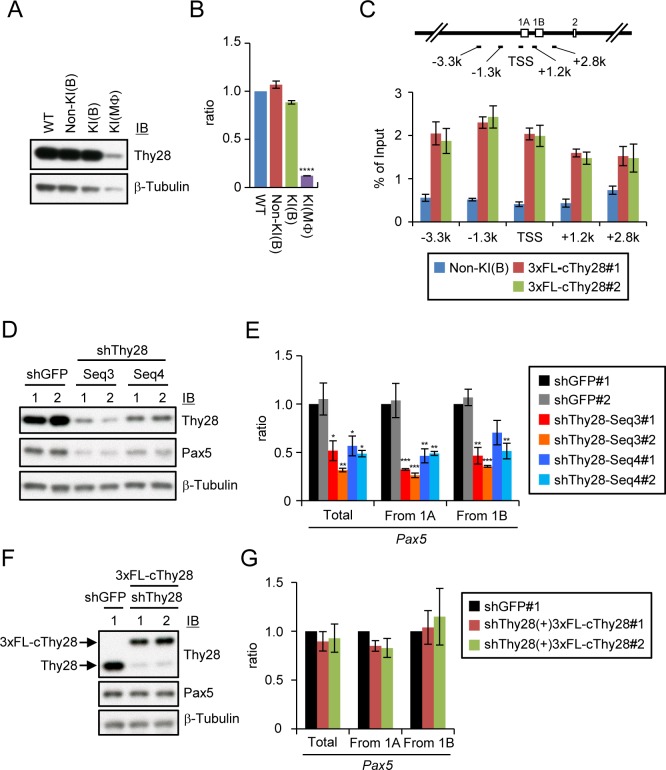
Critical roles of Thy28 associated with the *Pax5* 1A promoter in *Pax5* expression. **(A, B)** Expression of Thy28 protein **(A**) and *Thy28* mRNA **(B**) in DT40-derived cells. Expression levels of *Thy28* mRNA were quantified by real-time RT-PCR and normalized to those of *GAPDH* mRNA (mean +/− SEM, n = 3). **(C)** Interaction of Thy28 with the *Pax5* 1A promoter. The primer positions for real-time PCR are shown (upper panel). DT40 cell lines stably expressing 3xFLAG-tagged cThy28 or 3xFNLDD were used for ChIP with an anti-FLAG Ab (lower panel) (mean +/− SEM, n = 3). **(D, E)** shRNA-mediated knocking-down of Thy28 induced decrease in expression of Pax5 protein **(D)** and *Pax5* mRNA **(E)**. DT40 cell lines stably expressing shRNA against *GFP* or *Thy28* (Seq3 and Seq4) were analyzed. Expression levels of *Pax5* mRNA were quantified by real-time RT-PCR and normalized to those of *GAPDH* mRNA (mean +/− SEM, n = 3). p-values calculated between shGFP and shThy28 are shown (*: p < 0.05, **: p < 0.01, ***: p < 0.001). **(F, G)** RNAi rescue experiment. Expression of Pax5 protein **(F)** and *Pax5* mRNA **(G)** were analyzed with DT40 cell lines stably expressing shRNA against *Thy28* (Seq3) together with 3xFLAG-tagged silent mutant of cThy28. Expression levels of *Pax5* mRNA were quantified as described in **(E)** (mean +/− SEM, n = 3).

We found that expression of Thy28 is down-regulated in the macrophage-like cell lines trans-differentiated by ectopic expression of C/EBPβ (Fig. 6A, B). To confirm interaction of Thy28 with the *Pax5* 1A promoter, we performed ChIP analysis of 3xFLAG-tagged cThy28 expressed in DT40. As shown in Fig. 6C, 3xFLAG-tagged cThy28 interacted with the *Pax5* 1A promoter region. Binding of Thy28 to the *Pax5* locus could be detected at least up to −3.3 kbp and +2.8 kbp of the TSS of the exon 1A. This region contains both the exon 1A and 1B. Next, we examined the role of Thy28 in Pax5 expression. Down-regulation of Thy28 by shRNA led to decrease in expression of the Pax5 protein (Fig. 6D). shRNA-mediated knocking-down of Thy28 also down-regulated expression of *Pax5* transcripts using the exon 1A as well as the exon 1B (Fig. 6E), suggesting that Thy28 plays a role in transcription from both exons. We also examined expression of *AID* and IgM in Thy28 knocked-down cells. As shown in S1A Fig., *AID* expression was down-regulated in Thy28 knocked-down cells, consistent with a report that *AID* gene is a direct target of Pax5 [28]. In contrast, expression of IgM was not changed by down-regulation of Thy28 (S1B Fig.). These data suggest B cell identity was still maintained and argue against a possibility that Thy28 might be required for the proper maintenance of B cell identify, leading to down-regulation of Pax5 indirectly. Thus, the effects of Thy28 knocking-down on gene expression are specific to a set of genes, consistent with our idea that Thy28 directly regulates Pax5 expression. Expression of an shRNA-resistant form of cThy28 in cell lines, in which the endogenous Thy28 was knocked down, restored expression of Pax5 protein and mRNA (Fig. 6F, G, and S2 Fig.), suggesting that the effects of the used shRNA species are specific. These results indicated a critical role of Thy28 in the expression regulation of Pax5. Furthermore, these results showed that iChIP-SILAC can identify functional proteins interacting with an endogenous single-copy locus in vertebrate cells.

### Functional interaction of MYH9, a Thy28-interacting protein, with the *Pax5* 1A promoter

**Figure 7:**
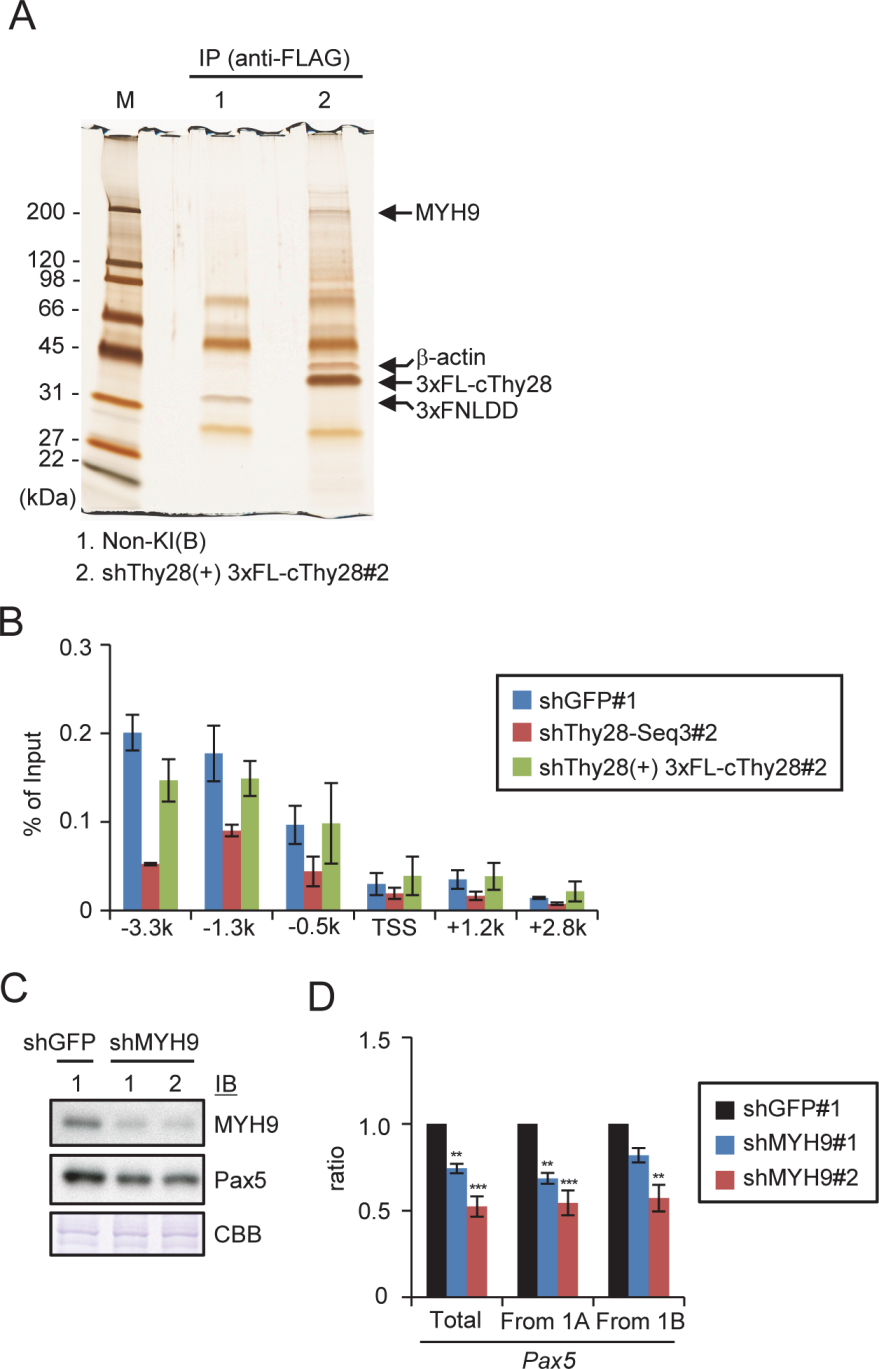
MYH9 recruited by Thy28 to the *Pax5* 1A promoter region induces Pax5 expression. **(A)** Identification of Thy28-interacting proteins. DT40-derived cell lines, 3xFNLDD and shThy28(+) 3xFL-cThy28#2, were used for immunoprecipitation with an anti-FLAG Ab. M: molecular weight markers. **(B)** Interaction of MYH9 with the *Pax5* 1A promoter region. DT40 cell lines, shGFP#1, shThy28-Seq3#2, shThy28(+)3xFL-cThy28#2, established in Fig. 6 were used for ChIP assay with an anti-MYH9 Ab (mean +/− SEM, n = 3). The primer positions for real-time PCR are shown in Fig. 6C. **(C, D)** shRNA-mediated knocking-down of MYH9 induced decrease in expression of Pax5 protein **(C)** and *Pax5* mRNA **(D)**. DT40 cell lines stably expressing shRNA against *GFP* or *MYH9* were analyzed. Coomassie Brilliant Blue (CBB) staining is shown as a protein loading control **(C)**. Expression levels of *Pax5* mRNA were quantified by real-time RT-PCR and normalized to those of *GAPDH* mRNA (mean +/− SEM, n = 3) **(D)**. p-values calculated between shGFP#1 and shMYH9 are shown (**: p < 0.01, ***: p < 0.001).

**Figure 8:**
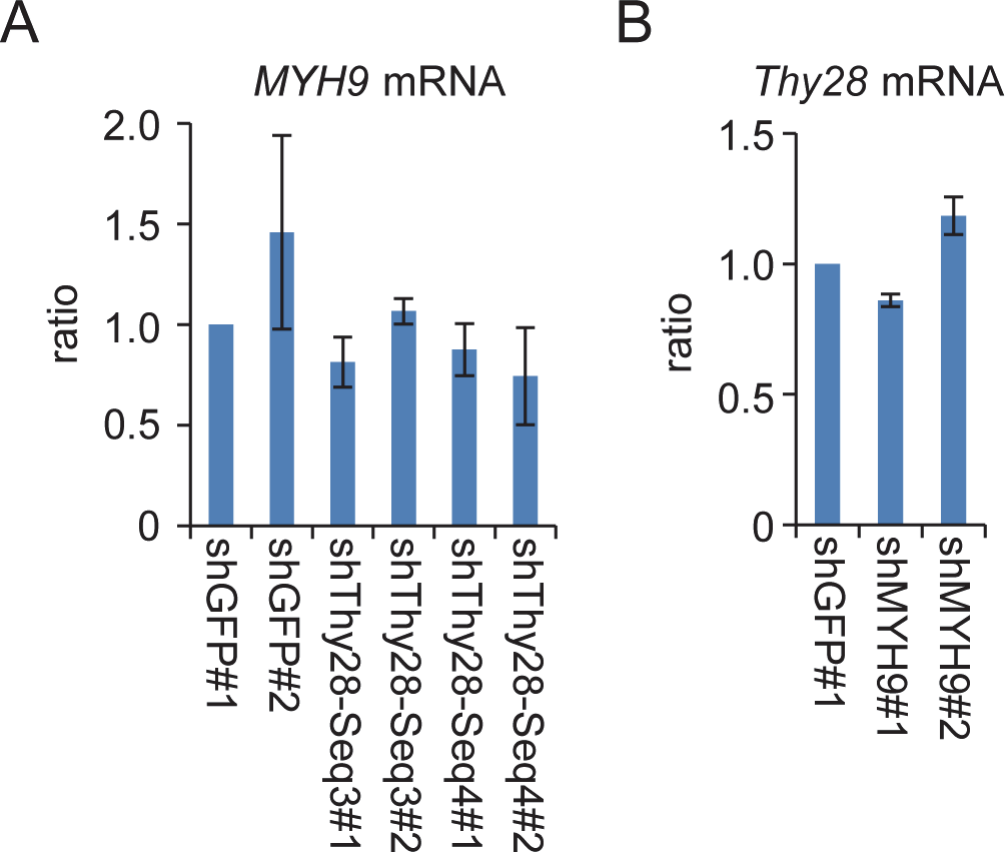
Expression of MYH9 or Thy28 in cell lines expressing shRNA. Total RNA was extracted from cell lines expressing shRNA and used in RT-PCR analysis. Expression levels of *MYH9* mRNA (**A**) or *Thy28* mRNA (**B**) were quantified by real-time RT-PCR and normalized to those of *GAPDH* mRNA (mean +/− SEM, n = 3).

Next, we attempted to identify proteins interacting with Thy28 to elucidate the molecular mechanisms, by which Thy28 induces Pax5 expression. We identified several proteins interacting with 3xFLAG-tagged cThy28 by immunoprecipitation using nuclear extracts prepared from a DT40-derived cell line expressing 3xFLAG-tagged cThy28 (Fig. 7A). Liquid chromatography coupled with tandem mass spectrometry (LC-MS/MS) revealed that the protein bands around 200 kDa and 45 kDa are MYH9 and β-actin, respectively (Fig. 7A). To examine potential involvement of MYH9 in transcription of the *Pax5* gene, we performed ChIP assay using anti-MYH9 Ab. As shown in Fig. 7B, we detected substantial binding of MYH9 to the *Pax5* 1A promoter region as well as modest interactions in the gene body in the parental DT40 cell and the control shGFP#1 cell, in which shRNA against *GFP* was expressed. The binding of MYH9 to the *Pax5* 1A promoter was impaired in the shThy28-Seq3#2 cell, in which Thy28 was knocked down, and restored in the shThy28(+) 3xFL-cThy28#2 cell, in which shRNA-resistant form of cThy28 was expressed under knocking-down of Thy28 (Fig. 7B). These results indicated that Thy28 mediates the recruitment of MYH9 to the *Pax5* 1A promoter region. Lastly, we knocked down MYH9 expression by shRNA to examine its role in *Pax5* transcription. Similar to knocking-down of Thy28, that of MYH9 induced decrease in expression of Pax5 protein and *Pax5* mRNA (Fig. 7C, D), suggesting that MYH9 plays an important role in transcription of the *Pax5* gene. It is of note that down-regulation of MYH9 did not affect Thy28 expression and *vice versa* (Fig. 8). These results collectively demonstrated that Thy28 is required for the recruitment of MYH9 to the *Pax5* 1A promoter region for transcription of the *Pax5* gene in chicken B cells.

## Discussion

In this study, we applied iChIP-SILAC to direct identification of proteins bound to the endogenous single-copy *Pax5* 1A promoter *in vivo*. Using 5 × 10^7^ cells, we could identify a list of candidate proteins interacting with the *Pax5* 1A promoter region (Tables 1 and S1 Table). Some proteins might bind directly to the promoter region of the *Pax5* gene for regulation of its expression. Other proteins might be present in the unidentified regulatory regions (e.g. distal enhancers or silencers), which interact with the *Pax5* 1A promoter, or in the genomic regions spatially proximal in the same chromosomal territory as well as transcription factory. It is noteworthy that iChIP-SILAC can be applicable to dissect an endogenous single-copy locus using only 5 × 10^7^ vertebrate cells. This high sensitivity will facilitate identification of components of chromatin in specific genomic regions.

By comparing B cells with trans-differentiated macrophage-like cells, a nuclear protein, Thy28, was found to be associated with the *Pax5* 1A promoter in a B cell-specific manner (Table 1 and Fig. 6). Thy28 is a protein conserved from bacteria to mammal [29]. Thy28 is highly expressed in bursa of Fabricius and lymphoid tissues in chicken [26]. Its expression is also detected in liver, heart and brain [26]. The highest expression in the bursa of Fabricius implies its important role for B cell development. In contrast to limited tissue distribution of cThy28, mouse Thy28 is more broadly expressed in various tissues such as thymus, brain, liver, kidney and testis [29], suggesting its species-specific roles. The N-terminal region of cThy28 protein (amino acid residues (a.a.) 1–71), which includes a nuclear localization signal (NLS), is not conserved among human and mouse, while the C-terminal region (a.a. 72–242) shows high homology [29]. It is of note that this conserved region shows conformational homology with the YTH domain, a potential RNA-binding domain, of YTH domain-containing protein 2 [30], suggesting its potential function through binding to RNA. Because Thy28 does not possess typical DNA-binding domains, it is possible that Thy28 may be recruited to the *Pax5* 1A promoter region through interaction with RNA such as non-coding RNA. We found that expression of Thy28 is down-regulated in the macrophage-like cell lines trans-differentiated by ectopic expression of C/EBPβ (Fig. 6A, B), suggesting that its expression is regulated in a B cell-specific manner. Our preliminary data showed that the binding of Thy28 decreases as the distance from the *Pax5* promoter increases. These data suggest that Thy28 binding might be specific to the *Pax5* promoter. However, at this stage, we cannot rule out the possibility that Thy28 may also bind to other genomic regions. This is an interesting future issue, and ChIP-Seq analysis of Thy28 would be informative. shRNA-mediated knocking-down of Thy28 led to down-regulation of Pax5 (Fig. 6D, E), indicating a critical role of Thy28 in the regulation of Pax5 expression. The effects of Thy28 knock-down were specific to a set of genes (Fig. 6E and S1 Fig.), consistent with the notion that Thy28 directly regulates expression of the *Pax5* gene. Although Thy28 is known to be involved in regulation of apoptosis [26,31,32], the link between functions of Thy28 in apoptosis and expression regulation of Pax5 is not clear at this stage.

**Figure 9:**
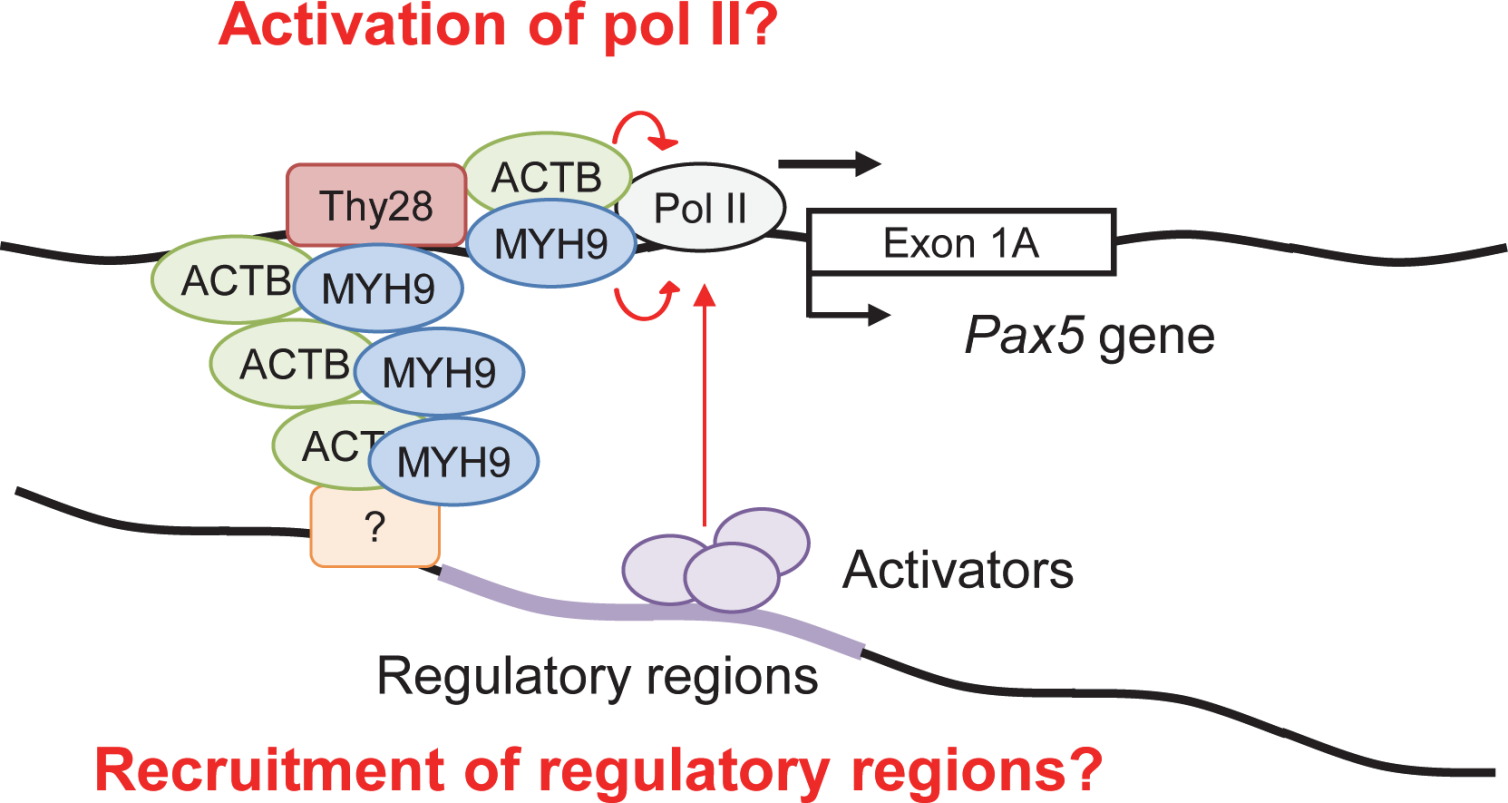
Potential mechanisms of expression regulation of *Pax5* gene mediated by the Thy28-MYH9 complex. MYH9 may directly regulate transcription of *Pax5* through regulation of transcriptional machinery. Alternatively, protein complexes including Thy28, β-actin (ACTB), and MYH9 may mediate inter- or intra-chromosomal interactions at the *Pax5* locus and other loci containing transcriptional regulatory regions. See the main text for details.

To elucidate molecular mechanisms how Thy28 regulates Pax5 expression, we identified proteins interacting with Thy28. By immunoprecipitation combined with mass spectrometric analysis, we identified β-actin and MYH9 as Thy28-interacting proteins (Fig. 7A). Although it is well known that the actin-myosin system is involved in intracellular transport as well as muscle contraction, their other functions have also been shown [33]. Especially, in addition to its typical roles in the cytoplasm, it has been reported that some family members of actin- and myosin-related proteins are localized in the nucleus [34], suggesting their function in the nucleus. Importantly, β-actin interacts with pol II and induces formation of transcriptional pre-initiation complexes for acceleration of transcription by pol II [35]. Therefore, it is possible that Thy28 recruits β-actin to the *Pax5* locus and/or enhances the transcriptional function of β-actin for *Pax5* transcription. MYH9 is a member of myosin superfamily of motor proteins, and its defect causes MYH9-related disease (MYH9-RD), an autosomal dominant thrombocytopenia with giant platelets [36]. Here, we showed that MYH9 is present in the *Pax5* 1A promoter region in the nucleus and involved in transcription of the *Pax5* gene (Fig. 7B–D). Additionally, Thy28 was required for the recruitment of MYH9 to the *Pax5* locus (Fig. 7B). Knocking-down of Thy28 or MYH9 down-regulated expression of the *Pax5* transcripts using the exon 1A as well as the exon 1B (Figs. 6D, E, 7C, D). Since binding of Thy28 to the *Pax5* locus could be detected not only in the promoter region of the exon 1A but also in that of the exon 1B (+1.2k in Fig. 6C), these results are consistent with the idea that Thy28 regulates expression of both transcripts using the exon 1A and the exon 1B. Different from the distribution pattern of Thy28 on the *Pax5* locus (Fig. 6C), MYH9 was mainly associated with the *Pax5* 1A promoter region (Fig. 7B). Therefore, the genomic region upstream of the *Pax5* exon 1A may include regulatory element controlled by MYH9 for transcription from the exon 1B, although we cannot eliminate the possibility that modest association of MYH9 with the genomic region upstream of the exon 1B (+1.2k in Fig. 7B) is sufficient for activation of transcription from the exon 1B. How does MYH9 regulate *Pax5* transcription? MYH9 may directly regulate transcription of *Pax5* through regulation of transcriptional machinery. It has been reported that nuclear myosin I (NMI) and myosin VI, members of myosin superfamily, are localized in the nucleus and directly regulate pol II-mediated transcription [34,35,37–39], suggesting a possibility that MYH9 induces *Pax5* transcription by similar mechanisms (Fig. 9). Alternatively, considering that it has been reported that nuclear complexes of β-actin and NMI mediate inter-chromosomal interactions [40], protein complexes including Thy28, β-actin, and MYH9 may mediate inter- or intra-chromosomal interactions at the *Pax5* locus and other loci containing transcriptional regulatory regions (Fig. 9). Elucidation of the molecular mechanisms how Thy28, β-actin, and MYH9 regulate *Pax5* transcription is an important future issue.

## Materials and Methods

### Cell culture

The chicken B cell line DT40 and DT40-derived cells which retain B cell phenotypes were grown as described previously [18]. DT40-derived cells trans-differentiated into macrophage-like cells were grown as described above with 5% chicken serum.

### Construction of the targeting vector

The nucleotide sequences 2.6 kbp upstream and 2.2 kbp downstream of the insertion site of LexA binding elements (BE) were amplified by PCR with DT40 genomic DNA as template. The 2.6 kbp fragment was inserted between *Xho* I and *Cla* I sites of pTNLrSP4 [41] to construct pTNL-cPax5-LongArm. LexA BE from 8xLexA-binding elements/pMD20 (#48807, Addgene) [2] and the 2.2 kbp fragment were inserted sequentially between *Sal* I and *Not* I sites of pTNL-cPax5-LongArm to construct pTNL-LexA-cPax5.

### Primers

The primers used in this study are shown in S2 Table.

### Gene targeting

For gene targeting, DT40 cells (1 × 10^7^ cells) were transfected with linearized pTNL-LexA-cPax5 (10 μg) by electroporation using Gene Pulser II (Bio-Rad) at 550 V, 25 μF. The transfected cells were cultured in the presence of G418 (2 mg/ml) in 48-well plates. The survived cell colonies were individually picked up and expanded as targeted cell lines. To eliminate the floxed neomycin resistance (Neo^r^) gene, the targeted cell lines were transfected with the GFP-fused Cre expression vector pCAG-Cre:GFP (#13776, Addgene) [42] under the same transfection condition. After single cell sorting, clones were expanded.

### Confirmation of insertion of LexA BE

The insertion of LexA BE into the *Pax5* promoter was confirmed by genomic PCR with KOD FX (Toyobo) according to the manufacturer’s instructions. The primer sets annealing with endogenous genomic regions and the inserted Neo^r^ gene are shown in S2 Table. The insertion was also confirmed by Southern blot analysis at PhoenixBio Co., Ltd. (Japan). Long and Short probes were amplified by PCR with primer sets (S2 Table). The nucleotide sequence of Neo probe is shown in S3 Fig.

### Expression of 3xFNLDD

To express 3xFNLDD, DT40-derived cells (1 × 10^7^ cells) were transfected with 100 μg of the linearized 3xFNLDD/pCMV-7.1 plasmid (#48874, Addgene) [3] together with 3 μg of the Neo^r^ by electroporation using Gene Pulser II (Bio-Rad) at 250 V, 950 μF and cultured in the presence of G418 (2 mg/ml). The survived cell colonies were individually picked up and expanded (KI(B) clones). One of the DT40-derived cells retaining LexA-binding elements (LexA BE) and expressing 3xFNLDD (KI(B) #4) was simply referred as KI(B) and used for iChIP analysis.

### Construction of the chicken C/EBPβ expression plasmid

Chicken C/EBPβ (cC/EBPβ) cDNA was amplified by RT-PCR with DT40 total RNA as template and cloned between *Hind* III and *Bam*H I sites of pEF vector [43] to construct cC/EBPβ/pEF. The primer sets used for amplification of cC/EBPβ are shown in S2 Table.

### Trans-differentiation of DT40 cells into macrophage-like cells

For trans-differentiation into macrophage-like cells, DT40-derived cells (1 × 10^7^ cells) were co-transfected with 110 μg of linearized cC/EBPβ/pEF and 3 μg of puromycin-resistance gene by electroporation. The transfected cells were selected in the presence of puromycin (0.35 μg/ml). The survived cell colonies were individually picked up and expanded with 5% chicken serum. One of the trans-differentiated macrophage-like cell lines retaining LexA BE and expressing 3xFNLDD was named as KI(MΦ) and used for iChIP analysis.

### Immunoblot analysis

Cells were lysed in high salt lysis buffer (20 mM Tris-HCl pH 8.0, 25% glycerol, 420 mM NaCl, 1.5 mM MgCl_2_, 0.2 mM EDTA, complete protease inhibitor cocktail without EDTA (Roche)) for 30 min on ice. After centrifugation (17,400 × g) at 4°C for 10 min, the supernatant was collected as whole cell lysate. Nuclear extracts were prepared with NE-PER Nuclear and Cytoplasmic Extraction Reagents (Thermo Fisher Scientific). Immunoblot analysis was performed with anti-FLAG M2 antibody (Ab) (Sigma-Aldrich), anti-Pax5 Ab (sc-1974, Santa Cruz Biotechnology), anti-C/EBPβ Ab (sc-150, Santa Cruz Biotechnology), anti-β-tubulin Ab (MMS-410P, Covance), anti-MYH9 Ab (sc-47199, Santa Cruz Biotechnology), and anti-chicken Thy28 (cThy28) Ab (kindly gifted by Dr. Compton) [26].

### Quantitative real-time RT-PCR and semi-quantitative RT-PCR

Quantitative real-time RT-PCR was performed with total RNA as described previously [44]. Semi-quantitative RT-PCR was performed as described previously [18] except for using AmpliTaq Gold 360 Master Mix (Applied Biosystems).

### Statistical analysis

Statistical analysis was performed with the Prism software 6 (GraphPad) using *t* test (Fig. 4B) or one-way analysis of variance (ANOVA) (other Figures).

### Flowcytometry

Cells were stained for 30 min at 4°C with R-phycoerythrin-conjugated anti-chicken IgM (SouthernBiotech, 8310-09). Flowcytometric analysis was performed with FACS Calibur (BD Biosciences) and analyzed with FlowJo software (TreeStar).

### iChIP-SILAC

KI(B) was grown at 39.5°C in RPMI-1640 and FCS provided in Pierce SILAC Protein Quantitation Kit-RPMI Kit (Thermo Fisher Scientific) with 4 mM glutamine, 1% chicken serum, 50 μM 2-mercaptoethanol, ^13^C_6_ L-Lysine-2HCl (Thermo Fisher Scientific), and ^13^C_6_^15^N_4_L-Arginine-HCl (Thermo Fisher Scientific) according to the manufacture’s instructions. KI(MΦ) were grown at 39.5°C in RPMI-1640 and FCS provided in Pierce SILAC Protein Quantitation Kit-RPMI Kit (Thermo Fisher Scientific) with 4 mM glutamine, 5% chicken serum, 50 μM 2-mercaptoethanol, Lysine-2HCl (Thermo Fisher Scientific), and L-Arginine-HCl (Thermo Fisher Scientific) according to the manufacture’s instructions. Isotopically labeled KI(B) (5 × 10^7^ cells) and non-labeled KI(MΦ) (5 × 10^7^ cells) were mixed and fixed with 1% formaldehyde at 37°C for 5 min. The chromatin fraction was extracted and fragmented by sonication (the average length of fragments was about 2 kbp) as described previously [45] except for using 4 ml of Sonication Buffer [2]. The sonicated chromatin in Sonication Buffer with 1% TritonX-100 was used for iChIP as described previously [2]. Briefly, the sonicated chromatin was pre-cleared with 75 μg of normal mouse IgG (Santa Cruz Biotechnology) conjugated to 750 μl of Dynabeads-Protein G (Invitrogen) and subsequently incubated with 75 μg of anti-FLAG M2 Ab (Sigma-Aldrich) conjugated to 750 μl of Dynabeads-Protein G at 4°C for 20 h. The Dynabeads were washed 2 times each with 1.5 ml of Low Salt Wash Buffer, High Salt Wash Buffer, and LiCl Wash Buffer, and once with 1.5 ml of TBS Buffer (50 mM Tris-HCl (pH 7.5), 150 mM NaCl) with 0.1% IGEPAL-CA630. The immunoprecipitants were eluted with 400 μl of Elution Buffer (500 μg/ml 3xFLAG peptide (Sigma-Aldrich), 50 mM Tris-HCl (pH 7.5), 150 mM NaCl, 0.1% IGEPAL-CA630) at 37°C for 20 min. The eluted chromatin complexes were precipitated in 1 ml of 2-propanol with 50 μl of 3M sodium acetate and 5 μl of 20 mg/ml glycogen at −20°C overnight. After centrifugation (17,400 × g) at 4°C for 30 min, the precipitants were washed with 1 ml of 70% ethanol and then incubated in 50 μl of 2 × Sample Buffer [2] at 98°C for 30 min for reverse-crosslinking and denaturation of proteins. The reverse-crosslinked proteins were subjected to SDS-PAGE and visualized by silver staining with Dodeca silver staining kit (Bio-Rad). After SDS-PAGE followed by silver staining, visualized proteins were excised and analyzed using a nanoLC-MS/MS system composed of LTQ Orbitrap Velos (Thermo Fisher Scientific) coupled with nanoLC (Advance, Michrom BioResources) and HTC-PAL autosampler (CTC Analytics) at DNA-chip Development Center for Infectious Diseases (RIMD, Osaka University). Data were acquired using Xcalibur software. Quantification was performed using Proteome discoverer 1.2 (Thermo Fisher Scientific) and Mascot search engine (Matrix Science) for peptide identification against the Swissprot database. The initial mass tolerance was set to 10 ppm, and MS/MS mass tolerance was 0.8 Da. Enzyme was set to trypsin/p with two missed cleavages. Carbamidomethylation of cysteine was searched as fixed modification, whereas N-acetyl-protein and oxidation of methionine were searched as variable modification. A minimum of two peptides was quantified for each protein. Raw Heavy/Light value was normalized to iChIP efficiency of the c*Pax5* 1A promoter region and shown as normalized value in Table 1.

### Construction of the chicken Thy28 expression plasmid

Chicken Thy28 (cThy28) cDNA was amplified by RT-PCR with DT40 total RNA as template and cloned between *Eco*R I and *Xba* I sites of p3xFLAG-CMV-7.1 vector (Sigma-Aldrich) to construct cThy28/p3xFLAG-CMV. The primer sets used for amplification of cThy28 are shown in S2 Table.

### Establishment of DT40-derived cells expressing 3xFLAG-tagged cThy28

For expression of 3xFLAG-tagged cThy28, DT40 cells (1 × 10^7^ cells) were co-transfected with 100 μg of linearized cThy28/p3xFLAG-CMV with 3 μg of Neo^r^ gene by electroporation. The transfected cells were selected in the presence of G418 (1.5 mg/ml). The survived cell colonies were individually picked up and cultured.

### ChIP

ChIP with anti-FLAG M2 Ab or anti-MYH9 Ab were performed as described previously [2]. The DNA was purified with ChIP DNA Clean & Concentrator (Zymo Research) and used as template for real-time PCR with SYBR Select PCR system (Applied Biosystems) using the Applied Biosystems 7900HT Fast Real-Time PCR System.

### Construction of knock-down plasmids

The Neo^r^ gene of pcDNA3.1(−)/*myc*-His A (Invitrogen) was amplified by PCR and inserted into the *Xho* I site of pSUPER (Oligoengine) to construct pSUPER-Neo. The following oligonucleotides were annealed and inserted between *Bgl* II and *Hind* III sites downstream of the H1 promoter in pSUPER-Neo to construct pSUPER-Neo-GFP, pSUPER-Neo-cThy28#3, pSUPER-Neo-cThy28#4, and pSUPER-Neo-cMYH9: GFP; forward oligonucleotide 5’-GATCCCCgcaagctgaccctgaagttTTCAAGAGAaacttcagggtcagcttgcTTTTTA-3’, reverse oligonucleotide 5’-AGCTTAAAAAgcaagctgaccctgaagttTCTCTTGAAaacttcagggtcagcttgcGGG-3’, cThy28#3; forward oligonucleotide 5’-GATCCCCgaacatgatgctcttctcaTTCAAGAGAtgagaagagcatcatgttcTTTTTA-3’, reverse oligonucleotide 5’-AGCTTAAAAAgaacatgatgctcttctcaTCTCTTGAAtgagaagagcatcatgttcGGG-3’, cThy28#4; forward oligonucleotide 5’-GATCCCCagaatctgattctggtggaTTCAAGAGAtccaccagaatcagattctTTTTTA-3’, reverse oligonucleotide 5’-AGCTTAAAAAagaatctgattctggtggaTCTCTTGAAtccaccagaatcagattctGGG-3’, cMYH9; forward oligonucleotide 5’- GATCCCCggatctggaaagccatataTTCAAGAGAtatatggctttccagatccTTTTTA-3’, reverse oligonucleotide 5’- AGCTTAAAAAggatctggaaagccatataTCTCTTGAAtatatggctttccagatccGGG-3’.

### Plasmids for RNAi rescue experiments

To generate an shRNA-resistant form of Thy28, silent mutation was introduced in the target sequence of shRNA cThy28#3 in cThy28 cDNA with QuikChange Site-Directed Mutagenesis Kit (Agilent Technologies). The primer sets used for the mutagenesis are shown in S2 Table. The DNA sequence of 3xFLAG-tagged silent mutant of cThy28 was amplified by PCR and cloned between *Nhe* I and *Xba* I sites of pcDNA3.1/Hygro(-) (Life Technologies) to construct pcDNA-Hyg-3xFL-cThy28-mut.

### Knocking-down experiments

For stable knocking-down, DT40 cells (1 × 10^7^ cells) were transfected with 20 μg of the linearized knocking-down vectors by electroporation and selected in the presence of G418 (1.5 mg/ml). The survived cell colonies were picked up individually and cultured. To examine whether expression of an shRNA-resistant form of cThy28 in cell lines, in which the endogenous Thy28 is knocked down, restores expression of Pax5 protein and mRNA, DT40 cells (1 × 10^7^ cells) were co-transfected with 100 μg of linearized pcDNA-Hyg-3xFL-cThy28-mut with 10 μg of pSUPER-Neo-cThy28#3 by electroporation. The transfected cells were selected in the presence of G418 (1.5 mg/ml). The survived cell colonies were individually picked up and cultured.

### Immunoprecipitation and mass spectrometric analysis

Nuclear extracts were prepared as described previously [46]. Nuclear extracts were pre-cleared with 6 μg of normal mouse IgG (Santa Cruz Biotechnology) conjugated to 60 μl of Dynabeads-Protein G (Invitrogen) and subsequently incubated with 6 μg of anti-FLAG M2 Ab conjugated to 60 μl of Dynabeads-Protein G at 4°C for 2 h. The Dynabeads were washed five times with 1 ml of Wash Buffer (20 mM Tris pH 8.0, 150 mM NaCl, 25% glycerol, 1.5 mM MgCl_2_, 0.2 mM EDTA, 0.1% IGEPAL-CA630) and once with 1 ml of TBS buffer with 0.1% IGEPAL-CA630. The immunoprecipitants were eluted with 40 μl of Elution Buffer, mixed with 2 × Sample Buffer, boiled for 5 min, and subjected to SDS-PAGE. The proteins visualized by silver staining were excised and analyzed by LC-MS/MS at DNA-chip Development Center for Infectious Diseases (RIMD, Osaka University).

## Supporting Information

S1 Fig Expression of *AID* and IgM in DT40 cell lines stably expressing shRNA against *GFP* or *Thy28*. (PDF)

S2 Fig RNAi rescue experiment. (PDF)

S3 Fig The nucleotide sequence of Neo probe used in Southern blot analysis. (PDF)

S1 Table Proteins identified by iChIP-SILAC. (XLSX)

S2 Table Primers used in this study. (PDF)

## References

[1] Hoshino A, Fujii H (2009) Insertional chromatin immunoprecipitation: a method for isolating specific genomic regions. J Biosci Bioeng 108: 446–449. 10.1016/j.jbiosc.2009.05.005 19804873

[2] Fujita T, Fujii H (2011) Direct idenification of insulator components by insertional chromatin immunoprecipitation. PLoS One 6: e26109 10.1371/journal.pone.0026109 22043306 PMC3197142

[3] Fujita T, Fujii H (2012) Efficient isolation of specific genomic regions by insertional chromatin immunoprecipitation (iChIP) with a second-generation tagged LexA DNA-binding domain. Adv Biosci Biotechnol 3: 626–629.

[4] Fujita T, Fujii H (2013) Locus-specific biochemical epigenetics / chromatin biochemistry by insertional chromatin immunoprecipitation. ISRN Biochem 2013: Article ID 913273. 10.1155/2013/913273 PMC4392943 25969763

[5] Fujita T, Fujii H (2013) Efficient isolation of specific genomic regions and identification of associated proteins by engineered DNA-binding molecule-mediated chromatin immunoprecipitation (enChIP) using CRISPR. Biochem Biophys Res Commun 439: 132–136. 10.1016/j.bbrc.2013.08.013 23942116

[6] Fujita T, Asano Y, Ohtsuka J, Takada Y, Saito K, et al. (2013) Identification of telomere-associated molecules by engineered DNA-binding molecule-mediated chromatin immunoprecipitation (enChIP). Sci Rep 3: 3171 10.1038/srep03171 24201379 PMC3821016

[7] Fujita T, Fujii H (2014) Identification of proteins associated with an IFNγ-responsive promoter by a retroviral expression system for enChIP using CRISPR. PLoS One 9: e103084 10.1371/journal.pone.0103084 25051498 PMC4106880

[8] Fujita T, Fujii H (2014) Efficient isolation of specific genomic regions retaining molecular interactions by the iChIP system using recombinant exogenous DNA-binding proteins. BMC Mol Biol 15: 26. 25428274 10.1186/s12867-014-0026-0 PMC4253623

[9] Harrison MM, Jenkins BV, O′Connor-Giles KM, Wildonger J (2014) A CRISPR view of development. Genes Dev 28: 1859–1872. 10.1101/gad.248252.114 25184674 PMC4197953

[10] Cobaleda C, Schebesta A, Delogu A, Busslinger M (2007) Pax5: the guardian of B cell identity and function. Nat Immunol 8: 463–470. 17440452 10.1038/ni1454

[11] Urbánek P, Wang ZQ, Fetka I, Wagner EF, Busslinger M (1994) Complete block of early B cell differentiation and altered patterning of the posterior midbrain in mice lacking Pax5/BSAP. Cell 79: 901–912. 8001127 10.1016/0092-8674(94)90079-5

[12] Nutt S, Urbánek P, Rolink A, M B (1997) Essential functions of Pax5 (BSAP) in pro-B cell development: difference between fetal and adult B lymphopoiesis and reduced V-to-DJ recombination at the IgH locus. Genes Dev 11: 476–491. 9042861 10.1101/gad.11.4.476

[13] Nutt SL, Neavey B, Rolink AG, Busslinger M (1999) Commitment to the B-lymphoid lineage depends on the transcription factor Pax5. Nature 401: 556–562. 10524622 10.1038/44076

[14] Schaniel C, Bruno L, Melchers F, Rolink AG (2002) Multiple hematopoietic cell lineages develop in vivo from transplanted Pax5-deficient pre-B I-cell clones. Blood 99: 472–478. 11781227 10.1182/blood.v99.2.472

[15] Decker T, Pasca di Magliano M, McManus S, Sun Q, Bonifer C, et al. (2009) Stepwise activation of enhancer and promoter regions of the B cell commitment gene Pax5 in early lymphopoiesis. Immunity 30: 508–520. 10.1016/j.immuni.2009.01.012 19345119

[16] O’Riordan M, Grosschedl R (1999) Coordinate regulation of B cell differentiation by the transcription factors EBF and E2A. Immunity 11: 21–31. 10435576 10.1016/s1074-7613(00)80078-3

[17] Ong SE, Biagoev B, Kratchmarova I, Kristensen DB, Steen H, et al. (2002) Stable isotope labeling by amino acids in cell culture, SILAC, as a simple and accurate approach to expression proteomics. Mol Cell Proteomics 1: 376–386. 12118079 10.1074/mcp.m200025-mcp200

[18] Fujita T, Fujii H (2011) Species-specific 5′-genomic structure and multiple transcription start sites in the chicken Pax5 gene. Gene 477: 24–31. 10.1016/j.gene.2011.01.008 21241785

[19] Buerstedde JM, Takeda S (1991) Increased ratio of targeted to random integration after transfection of chicken B cell lines. Cell 67: 179–188. 1913816 10.1016/0092-8674(91)90581-i

[20] Xie H, Ye M, Feng R, Graf T (2004) Stepwise reprogramming of B cells into macrophages. Cell 117: 663–676. 15163413 10.1016/s0092-8674(04)00419-2

[21] Kurosaki T (1999) Genetic analysis of B cell antigen receptor signaling. Annu Rev Immunol 17: 555. 10358768 10.1146/annurev.immunol.17.1.555

[22] Yang WC, Ghiotto M, Castellano R, Collette Y, Auphan N, et al. (2000) Role fo Tec kinase in nuclear factor of activated T cells signaling. Int Immunol 12: 1547. 11058574 10.1093/intimm/12.11.1547

[23] Webb CF, Yamashita Y, Ayers N, Evetts S, Paulin Y, et al. (2000) The transcription factor *Bright* associates with Bruton′s tyrosine kinase, the defective protein in immunodeficiency disease. J Immunol 165: 6956–6965. 11120822 10.4049/jimmunol.165.12.6956

[24] Hirano M, Kikuchi Y, Nisitani S, Yamaguchi A, Satoh A, et al. (2004) Bruton’s tyrosine kinase (Btk) enhances transcriptional co-activation activity of BAM11, a Btk-associated molecule of a subunit of SWI/SNF complexes. Int Immunol 16: 747–757. 15096481 10.1093/intimm/dxh076

[25] Munemasa Y, Suzuki T, Aizawa K, Miyamoto S, Imai Y, et al. (2008) Promoter region-specific histone incorporation by the novel histone chaperon ANP32B and DNA-binding factor KLF5. Mol Cell Biol 28: 1171–1181. 18039846 10.1128/MCB.01396-07 PMC2223403

[26] Compton MM, Thomson JM, Icard AH (2001) The analysis of cThy28 expression in avian lymphocytes. Apoptosis 6: 299–314. 11445672 10.1023/a:1011339626128

[27] Chen CM, Cepko CL (2000) Expression of Chx10 and Chx10-1 in the developing chicken retina. Mech Dev 90: 293. 10640715 10.1016/s0925-4773(99)00251-8

[28] Gonda H, Sugai M, Nambu Y, Katakai T, Agata Y, et al. (2003) The balance between Pax5 and Id2 activities is the key to AID gene expression. J Exp Med 198: 1427–1437. 14581609 10.1084/jem.20030802 PMC2194241

[29] Miyaji H, Yoshimoto T, Asakura H, Komachi A, Kamiya S, et al. (2002) Molecular cloning and characterization of the mouse thymocyte protein gene. Gene 297: 189–196. 12384300 10.1016/s0378-1119(02)00886-7

[30] Yu F, Song A, Xu C, Sun L, Li J, et al. (2009) Determining the DUF55-domain structure of human thymocyte nuclear protein 1 from crystals partially twinned by tetartohedry. Acta Crystallogr D Biol Crystallogr 65: 212–219. 19237743 10.1107/S0907444908041474

[31] Jiang XX, Toyota T, Yoshimoto T, Takada E, Asakura H, et al. (2003) Anti-IgM-induced down-regulation of nuclear Thy28 protein expression in Ramos B lymphoma cells. Apoptosis 8: 509. 14601557 10.1023/a:1025594409056

[32] Toyota H, Jiang XZ, Asakura H, Mizuguchi J (2012) Thy28 partially prevents apoptosis induction following engagement of membrane immunoglobulin in WEHI-231 B lymphoma cells. Cell Mol Biol Lett 17: 36. 22139584 10.2478/s11658-011-0034-8 PMC6275998

[33] Woolner S, Bement WM (2009) Unconventional myosins acting unconventionally. Trends Cell Biol 19: 245–252. 19406643 10.1016/j.tcb.2009.03.003 PMC4878029

[34] de Lanerolle P, Serebryannyy L (2011) Nuclear actin and myosins: life without filaments. Nat Cell Biol 13: 1282–1288. 22048410 10.1038/ncb2364

[35] Hofmann WA, Stojiljkovic L, Fuchsova B, Vargas GM, Mavrommatis E, et al. (2004) Actin is part of pre-initiation complexes and is necessary for transcription by RNA polymerase II. Nat Cell Biol 6: 1094–1101. 15502823 10.1038/ncb1182

[36] Balduini CL, Pecci A, Savoia A (2011) Recent advances in the understanding and management of MYH9-related inherited thrombocytopenias. Br J Haematol 154: 161–174. 21542825 10.1111/j.1365-2141.2011.08716.x

[37] Pestic-Dragovich L, Stojiljkovic L, Philimonenko AA, Nowak G, Ke Y, et al. (2000) A myosin I isoform in the nucleus. Science 290: 337–341. 11030652 10.1126/science.290.5490.337

[38] Hofmann WA, Vargas GM, Ramchandran R, Stojiljkovic L, Goodrich JA, et al. (2006) Nuclear myosin I is necessary for the formation of the first phosphodiester bond during transcription initiation by RNA polymerase II. J Cell Biochem 99: 1001–1009. 16960872 10.1002/jcb.21035

[39] Vreugde S, Ferrai C, Miluzio A, Hauben E, Marchisio PC, et al. (2006) Nuclear myosin VI enhances RNA polymerase II-dependent transcription. Mol Cell 23: 749–755. 16949370 10.1016/j.molcel.2006.07.005

[40] Hu Q, Kwon YS, Nunez E, Cardamone MD, Hutt KR, et al. (2008) Enhancing nuclear receptor-induced transcription requires nuclear motor and LSD1-dependent gene networking in interchromatin granules. Proc Natl Acad Sci U S A 105: 19199–19204. 19052240 10.1073/pnas.0810634105 PMC2593616

[41] Saint Fleur S, Hoshino A, Kondo K, Egawa T, Fujii H (2009) Regulation of Fas-mediated immune homeostasis by an activation-induced protein, Cyclon. Blood 114: 1355–1365. 19528538 10.1182/blood-2008-11-189118 PMC2727414

[42] Matsuda T, Cepko CL (2007) Controlled expression of transgenes introduced by in vivo electroporation. Proc Natl Acad Sci USA 104: 1027. 17209010 10.1073/pnas.0610155104 PMC1764220

[43] Fujii H, Nakagawa Y, Schindler U, Kawahara A, Mori H, et al. (1995) Activation of Stat5 by interleukin 2 requires a carboxyl-terminal region of the interleukin 2 receptor β chain but is not essential for the proliferative signal transmission. Proc Natl Acad Sci USA 92: 5482–5486. 7777534 10.1073/pnas.92.12.5482 PMC41719

[44] Fujita T, Fujii H (2012) Transcription start sites and usage of the first exon of mouse Foxp3 gene. Mol Biol Rep 39: 9613–9619. 22723000 10.1007/s11033-012-1825-3

[45] Fujita T, Ryser S, Tortola S, Piuz I, Schlegel W (2007) Gene-specific recruitment of positive and negative elongation factors during stimulated transcription of the MKP-1 gene in neuroendocrine cells. Nucleic Acids Res 35: 1007–1017. 17259211 10.1093/nar/gkl1138 PMC1807974

[46] Fujita T, Ryser S, Piuz I, Schlegel W (2008) Up-regulation of P-TEFb by the MEK1-extracellular signal-regulated kinase signaling pathway contributes to stimulated transcription elongation of immediate early genes in neuroendocrine cells. Mol Cell Biol 28: 1630–1643. 18086894 10.1128/MCB.01767-07 PMC2258797

