# Supplementary material for: A Critical Role of the Thy28-MYH9 Axis in B Cell-Specific Expression of the Pax5 Gene in Chicken B Cells
Source: PLoS One. 2015 Jan 21;10(1):e0116579. doi: 10.1371/journal.pone.0116579 (PMC4301804; doi:10.1371/journal.pone.0116579)
Supplement: S1 Fig — (PDF) [file pone.0116579.s001.pdf]

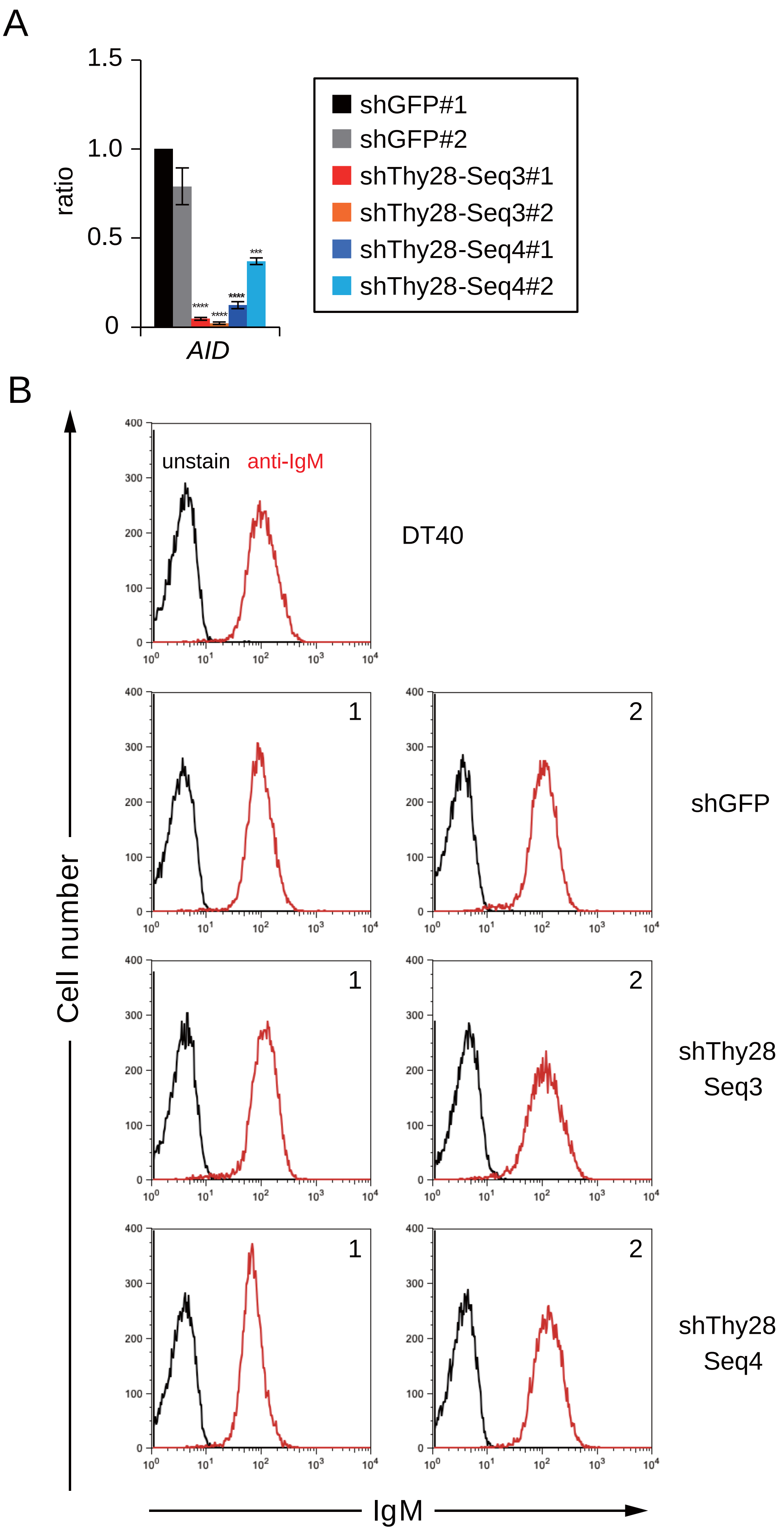
A
1.5
1.0
0.5
0
ratio
AID
****
****
****
***
shGFP#1
shGFP#2
shThy28-Seq3#1
shThy28-Seq3#2
shThy28-Seq4#1
shThy28-Seq4#2
B
Cell number
unstain
anti-IgM
DT40
1
2
shGFP
shThy28
Seq3
shThy28
Seq4
IgM

Figure S1. Expression of *AID* and IgM in DT40 cell lines stably expressing shRNA against *GFP* or *Thy28*. (A) Expression levels of *AID* mRNA were quantified by real-time RT-PCR and normalized to those of *GAPDH* mRNA (mean +/- SEM, n=3). DT40 cell lines stably expressing shRNA against *GFP* or *Thy28* (Seq3 and Seq4) were analyzed. p-values calculated between shGFP and shThy28 are shown (***: $p < 0.0001$, ****: $p < 0.0001$). **(C)** Flowcytometric analysis of expression of cell surface IgM.
