# Supplementary material for: A Critical Role of the Thy28-MYH9 Axis in B Cell-Specific Expression of the Pax5 Gene in Chicken B Cells
Source: PLoS One. 2015 Jan 21;10(1):e0116579. doi: 10.1371/journal.pone.0116579 (PMC4301804; doi:10.1371/journal.pone.0116579)
Supplement: S2 Fig — (PDF) [file pone.0116579.s002.pdf]

Fujita et al., Figure S2

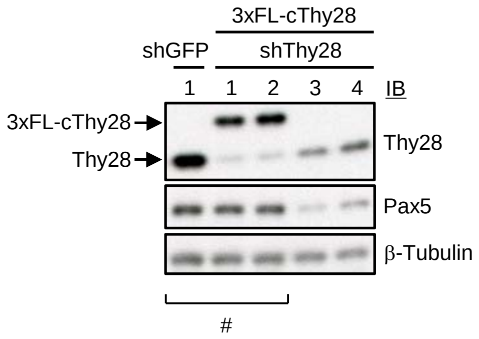

Figure S2. RNAi rescue experiment. Expression of Pax5 protein was analyzed with DT40 cell lines stably expressing shRNA against *Thy28* (Seq3) together with 3xFLAG-tagged silent mutant of cThy28. Undetectable expression of 3xFLAG-tagged silent mutant of cThy28 in DT40 cell lines stably expressing shRNA against *Thy28* failed to restore expression of Pax5 protein. #: The images are also shown in Figure 6F.
