# Supplementary material for: A Critical Role of the Thy28-MYH9 Axis in B Cell-Specific Expression of the Pax5 Gene in Chicken B Cells
Source: PLoS One. 2015 Jan 21;10(1):e0116579. doi: 10.1371/journal.pone.0116579 (PMC4301804; doi:10.1371/journal.pone.0116579)
Supplement: S3 Fig — (PDF) [file pone.0116579.s003.pdf]

Fujita et al., Figure S3

GAATTGATCCCCTCAGAAGAACTCGTCAAGAAGGCGATAGAAGGCGATGCGCT
GCGAATCGGGAGCGGCGATACCGTAAAGCACGAGGAAGCGGTCAGCCCATTC
GCCGCCAAGCTCTTCAGCAATATCACGGGTAGCCAACGCTATGTCCTGATAGC
GATCCGCCACACCCAGCCGGCCACAGTCGATGAATCCAGAAAAGCGGCCATTT
TCCACCATGATATTCGGCAAGCAGGCATCGCCATGGGTCACGACGAGATCCTC
GCCGTCGGGCATGCGCGCCTTGAGCCTGGCGAACAGTTCGGCTGGCGCGAGC
CCCTGATGCTCTTCGTCCAGATCATCCTGATTGACAAGACCGGCTTCCATCCGA
GTACGTGCTCGCTCGATGCGATGTTTCGCTTGGTGGTCGAATGGGCAGGTAGC
CGGATCAAGCGTATGCAGCCGCCGCATTGCATCAGCCATGATGGATACTTTCT
CGGCAGGAGCAAGGTGAGATGACAGGAGATCCTGCCCCGGCACTTCGCCCAA
TAGCAGCCAGTCCCTTCCCGCTTCAGTGACAACGTCGAGCA

Figure S3. The nucleotide sequence of Neo probe used in Southern blot analysis.
