# Supplementary material for: A Critical Role of the Thy28-MYH9 Axis in B Cell-Specific Expression of the Pax5 Gene in Chicken B Cells
Source: PLoS One. 2015 Jan 21;10(1):e0116579. doi: 10.1371/journal.pone.0116579 (PMC4301804; doi:10.1371/journal.pone.0116579)
Supplement: S2 Table — (PDF) [file pone.0116579.s005.pdf]

Table S2. Primers used in this study

Number	Name	Sequence (5' → 3')	Experiments
26554	cPax5-XhoCla-F	gactcgaggctaatggaaacagactgcttggtg	Cloning of the 2.6 kbp region upstream of the LexA BE insertion site for construction of the targeting vector
26556	cPax5-XhoCla-R	gcatcgattcacggccgagagaaggagg	Cloning of the 2.6 kbp region upstream of the LexA BE insertion site for construction of the targeting vector
26557	cPax5-SalSal-F	gggtcgaccacgcagcgcggggaggggtaacg	Cloning of the 2.2 kbp region downstream of the LexA BE insertion site for construction of the targeting vector
26562	cPax5-HindNot-R	tccgtccgcagtgcggccgcggtgatgccg	Cloning of the 2.2 kbp region downstream of the LexA BE insertion site for construction of the targeting vector
26632	cC/EBPb-S	ataagcttatgcaacgcctggtggcctgggacgca	Cloning of chicken C/EBPβ
26633	cC/EBPb-A	ttggatcctcagcagcggggcgaggaagcgagcag	Cloning of chicken C/EBPβ
27165	cTHYN1-EcoXba-F	aggaattcaatgccctggccgagcagaaagagggac	Cloning of chicken Thy28
27166	cTHYN1-EcoXba-R	tctctagattaatgtggcttttcctcttccaggctc	Cloning of chicken Thy28
26612	pTNL-cPax5XX2-F	gcgtttccaagcacattcacac	Genomic PCR 1 and 3 in Fig. 2
26613	pTNL-cPax5XX2-R	aggccagaggccacttgtgtag	Genomic PCR 1 in Fig. 2
26734	pTNL-cPax5HN-F	cgagatcagcagcctctgttccacatacac	Genomic PCR 2 in Fig. 2
26735	pTNL-cPax5HN-R	gcgagttgcgggaacttgtggcggctg	Genomic PCR 2 in Fig. 2
26558	cPax5-SalSal-R	ggtcagaataagtcgacccttccgctc	Genomic PCR 3 in Fig. 2
26745	cPax5-Probe7-F	gatgagcttgtgaagatgacaca	Cloning of the probe (Long) for southern blotting in Fig. 2
26746	cPax5-Probe7-R	ctcaccacctcaccagattctaa	Cloning of the probe (Long) for southern blotting in Fig. 2
26749	cPax5-Probe9-F	gagagtggagcgctttgtatg	Cloning of the probe (Short) for southern blotting in Fig. 2
26750	cPax5-Probe9-R	cttttaaaccaaagccaggaaat	Cloning of the probe (Short) for southern

			blotting in Fig. 2
26711	cPax5-ex3_4-F	aaattgcagagtacaaacgccaaaat	Quantitative RT-PCR (Pax5-All) in Figs. 3, 4, 6, and 7
26712	cPax5-ex3_4-R	acctttgtccgtatgatcctgttaat	Quantitative RT-PCR (Pax5-All) in Figs. 3, 4, 6, and 7
26538	cPax5-ex1A-F2	atcgcaatggatttggagaaga	Quantitative RT-PCR (Pax5-From 1A) in Figs. 3, 4, 6, and 7
26541	cPax5-ex1B-F4	ctgtaagcacgacccgtttg	Quantitative RT-PCR (Pax5-From 1A) in Figs. 3, 4, 6, and 7
26542	cPax5-ex2-R2	ggcggccattcacaaaaac	Quantitative RT-PCR (Pax5-From 1A and -From 1B) in Figs. 3, 4, 6, and 7
26886	cAID-F4	ctgagagagaacccagctgacat	Quantitative RT-PCR (AID) in Figs. 3 and 4
26887	cAID-R4	ggagcatgatgtagcactgtcac	Quantitative RT-PCR (AID) in Figs. 3 and 4
26547	cGAPDH-Ex8_9-F	agctgaatgggaagcttactgg	Quantitative RT-PCR (GAPDH) in Figs. 3, 4, 6-8 (used for normalization)
26548	cGAPDH-Ex8_9-R	agcagccttcactaccctcttg	Quantitative RT-PCR (GAPDH) in Figs. 3, 4, 6-8 (used for normalization)
26729	cM-CSFR_F	cgctgctcgggagagtcagaa	Semi-quantitative RT-PCR (M-CSFR) in Fig. 4
26730	cM-CSFR_R	gttggttccaggtgagagctt	Semi-quantitative RT-PCR (M-CSFR) in Fig. 4
27207	cTHYN1-Ex2_3-F	gcttcttggtgaagacaattcaaag	Quantitative RT-PCR (Thy28) in Figs. 6 and 8
27209	cTHYN1-Ex2_3-R	cctccttatcttcagtctcctcctc	Quantitative RT-PCR (Thy28) in Figs. 6 and 8
26895	cPax5-ChIP-LexA-DOWN-F	cgcacaggacatgatctcacac	iChIP and ChIP assays in Figs. 3, 6, and 7 (TSS)
26896	cPax5-ChIP-LexA-DOWN-R	ctgaaactaaacgttttaggtggaaa	iChIP and ChIP assays in Figs. 3, 6, and 7 (TSS)
26897	cPax5-ChIP-DOWN(+3k)-F	catacactccgaaaggaaaccac	iChIP and ChIP assays in Figs. 3, 6, and 7 (+2.8k)
26898	cPax5-ChIP-DOWN(+3k)-R	tttaatgaatgcgcatcaaaatg	iChIP and ChIP assays in Figs. 3, 6, and 7 (+2.8k)
27011	cPax5-ChIP-	ggagttatggaaaccaaagcacag	iChIP and ChIP assays in Figs. 3, 6, and 7 (-

	DOWN(-3k)-F		3.3k)
27012	cPax5-ChIP-DOWN(-3k)-R	atctcaatacatttcacacccaacc	iChIP and ChIP assays in Figs. 3, 6, and 7 (-3.3k)
27126	cPax5-ChIP-DOWN(+1k)-F	agcagcacactacggacaaactt	ChIP assays in Fig. 6 and 7 (+1.2k)
27127	cPax5-ChIP-DOWN(+1k)-R	gcgtacatcttctccaaatccat	ChIP assays in Fig. 6 and 7 (+1.2k)
27132	cPax5-ChIP-UP(-1k)-F3	attgtctagagacccttccagctc	ChIP assays in Fig. 6 and 7 (-1.3k)
27133	cPax5-ChIP-UP(-1k)-R3	gtctctccaggaaaagaagtgctc	ChIP assays in Fig. 6 and 7 (-1.3k)
27134	cPax5-ChIP-UP(-0.2k)-F	gggctcttatttcgtttttcttgtt	ChIP assays in Fig. 7 (-0.5k)
27135	cPax5-ChIP-UP(-0.2k)-R	gtgcttatttgtcagcgtggttg	ChIP assays in Fig. 7 (-0.5k)
27205	cTHYN1-3-mut-F	gcccctaaagaatatgatgctgttctcgagacaacgtctt	Silent mutation in chicken Thy28 cDNA
27206	cTHYN1-3-mut-R	aagacgttgtctcgagaacagcatcatattctttaggggc	Silent mutation in chicken Thy28 cDNA
